# Very High vs. High Tumor Mutational Burden Across Tumors: Real-World Associations with MSI, Pathway Features, and Immunotherapy Outcomes

**DOI:** 10.3390/biomedicines14030593

**Published:** 2026-03-06

**Authors:** Maria Fernanda Teixeira, Victoria Tomaz, Lucas Campos Barbosa e Silva, Uelson Donizete, Francisco Tustumi, Helder Imoto Nakaya, Juliana Rodrigues Beal, Fernando Moura, Mitesh J. Borad, Paulo Vidal Campregher, Pedro Luiz Serrano Uson Junior

**Affiliations:** 1 Center for Personalized Medicine, Hospital Israelita Albert Einstein, São Paulo 05652900, Brazil; maria.teixeira@einstein.br (M.F.T.); helder.nakaya@einstein.br (H.I.N.);; 2 Mayo Clinic Cancer Center, Phoenix, AZ 85054, USA; 3 Genesis Genomics, São Paulo 04703901, Brazil

**Keywords:** tumor mutational burden, immune checkpoint inhibitors, microsatellite instability, biomarkers, pathway analysis, precision oncology

## Abstract

**Background:** Tumor mutational burden (TMB) is an FDA-approved biomarker for immune checkpoint inhibitor (ICI) therapy. However, its predictive value varies among tumor types and molecular contexts. We investigated whether a very high TMB identifies a biologically distinct subset and whether a higher cutoff provides additional clinical insights beyond the conventional high TMB threshold. **Methods:** We analyzed 133 patients with advanced solid tumors and TMB ≥ 10 mutations/Mb (mut/Mb) who underwent tumor genomic profiling using a 523-gene DNA/RNA next-generation sequencing panel. Tumors were stratified into prespecified TMB categories: 10–20 mut/Mb (TMB-H) and >20 mut/Mb (TMB-VH). The clinical characteristics, ICI outcomes (in the treated subset), and pathway-level genomic features were compared between groups. **Results:** TMB-VH was observed in 42/133 (31.6%) patients and spanned more than 20 tumor types. MSI was markedly more prevalent in TMB-VH than in TMB-H tumors (38.1% vs. 2.2%; Fisher’s exact *p* = 8.9 × 10^−8^). Pathway-level comparisons did not identify statistically significant differences after false discovery rate correction (all *q* > 0.05), and the observed patterns were descriptive in nature. In the ICI-treated subset with complete follow-up, objective response did not differ according to the TMB group. Overall survival (OS) was also similar between groups, whether measured from metastatic diagnosis (log-rank *p* = 0.937) or from ICI initiation (log-rank *p* = 0.814), although OS was numerically longer in the TMB-VH group in both analyses without reaching statistical significance. **Conclusions:** In this cohort study, TMB-VH was strongly associated with MSI but not independently associated with improved ICI outcomes. Larger multicenter cohorts are needed to validate pathway-oriented patterns and clarify the clinical utility of extreme TMB thresholds across various histologies. Integrating the functional context (e.g., MSI status, gene-level context, and pathway-level features) with TMB magnitude may enable more robust, tumor-aware biomarker models for immunotherapy selection.

## 1. Introduction

TMB, broadly defined as the number of somatic variants per coding area of the tumor genome, has emerged as a promising biomarker for predicting the response to immune checkpoint blockade in multiple cancer types [1]. The biological rationale is intuitive: a higher mutational load increases the likelihood of neoantigen formation, which may enhance tumor immunogenicity and increase visibility to the immune system [2]. Clinically, this association has been most consistent in malignancies such as melanoma, non-small cell lung cancer, and urothelial carcinoma, where a higher TMB has been linked to improved outcomes with immunotherapy. However, this relationship is not uniform across tumor types, and some cancers do not show a clear correlation between neoantigen load and CD8+ T-cell infiltration [3,4].

KEYNOTE-158, a multicohort single-arm phase II trial, reported a higher objective response rate with pembrolizumab in TMB-H tumors (≥10 mut/Mb by FoundationOne CDx) than in non-TMB-H tumors (29% vs. 6%) [5]. Although the non-randomized design limits definitive conclusions, these findings support the FDA’s tumor-agnostic approval of pembrolizumab for TMB-H solid tumors [6].

Despite its regulatory approval, the broad adoption of TMB as a tumor-agnostic biomarker remains challenging. Technical variability across platforms and thresholds, along with an incomplete understanding of the biological determinants of immune sensitivity, continue to limit consistent clinical interpretation [7,8]. The relationship between TMB and immune responsiveness is far from linear and appears to be highly context-dependent [9]. TMB-VH thresholds have been explored in previous pan-cancer studies. However, the distinctive contribution of the present study is a pathway-level view of tumor biology, focusing on metabolic and signaling programs that co-occur with extreme hypermutation.

There is a growing need to complement somatic variant counts with a more mechanistic understanding of how TMB aligns with tumor-intrinsic biology and the immune microenvironment. The mutation load may reflect broader genomic instability and coexisting features such as immune infiltration, antigen presentation capacity, and tumor metabolism. While metabolic reprogramming is increasingly recognized as a determinant of immunogenicity and immune evasion [10,11], the interplay between these programs and extreme TMB remains underexplored. Our primary objective was to compare immunotherapy outcomes and pathway-level genomic profiles between tumors with TMB-H and TMB-VH in a real-world cohort. The secondary objective was to describe the gene-level context associated with TMB-VH values.

## 2. Methods

### 2.1. Study Design and Population

This retrospective, single-center study was conducted at a tertiary cancer center. We screened all patients with advanced or metastatic tumors who underwent tumor genomic profiling using next-generation sequencing (NGS) at our institution between March 2017 and August 2023. Patients with TMB ≥ 10 mut/Mb were eligible for the molecular analyses. For clinical outcome analyses, we included a subset of patients with metastatic disease who received at least one dose of ICI and had complete clinical follow-up data available for survival analysis. Follow-up was conducted until January 2025.

### 2.2. Clinical Data Collection

Clinical variables (age, sex, self-reported race, primary diagnosis/histology, stage at diagnosis, prior therapies, and ICI regimen details) were abstracted from electronic medical records. Staging followed the American Joint Committee on Cancer (AJCC) 8th edition.

### 2.3. Genomic Profiling and Biomarker Definitions

Tumor profiling was performed using a targeted DNA- and RNA-based next-generation sequencing (NGS) panel covering 523 genes (approximately 1.28 Mb in length). Sequencing was performed on an Illumina platform using a clinical laboratory-validated workflow. Patients were stratified a priori into TMB categories for analysis: 10–20 mut/Mb (TMB-H) and >20 mut/Mb (TMB-VH). TMB and microsatellite instability (MSI) were derived from a hybrid capture tumor-only NGS assay using a laboratory-validated bioinformatic pipeline. Germline testing results were recorded when available but were not routinely performed for all patients.

#### 2.3.1. TMB Calculation

TMB was reported as mut/Mb and calculated as the number of eligible somatic variants divided by the effective panel size. Eligible variants consisted of coding-region single-nucleotide variants (SNVs) and small insertions/deletions (indels), excluding multi-nucleotide variants (MNVs). Variants were required to meet the minimum technical thresholds (variant allele fraction ≥ 5% and coverage ≥ 50×) and pass laboratory quality filters.

Common germline polymorphisms were removed using population databases and additional post-database allele frequency/proximity filtering rules that were implemented in the software. Specifically, variants with an observed allele count ≥ 10 in any of the gnomAD exome, gnomAD genome, or 1000 Genomes were excluded, along with additional allele frequency/proximity rules, as described in the software documentation. Variants with a COSMIC count ≥ 50 were excluded from the numerator. Somatic status and clinical classification followed the institutional laboratory workflow aligned with AMP/ASCO/CAP standards. For TMB computation, variants were counted irrespective of the clinical tier (including variants of unknown significance), consistent with tumor-agnostic TMB definitions. The effective panel size denominator was defined as the total coding territory with coverage > 50×, after excluding low-confidence regions in which variants were not called.

#### 2.3.2. MSI Assessment

MSI status was derived from the same tumor-only NGS data using the assay’s MSI module. This approach evaluates a set of microsatellite loci for instability relative to a baseline set of normal samples using information entropy metrics and generates a sample-level score reflecting the proportion of unstable sites among the assessed loci. The MSI classification threshold and baseline normal set were assay-configurable parameters established during laboratory validation and were not available in clinical reports. Therefore, MSI was analyzed as reported by the clinical laboratory (MSI-positive/MSI-H vs. MSS) without applying an investigator-defined cutoff.

### 2.4. Pathway Mapping

**Figure 1:**
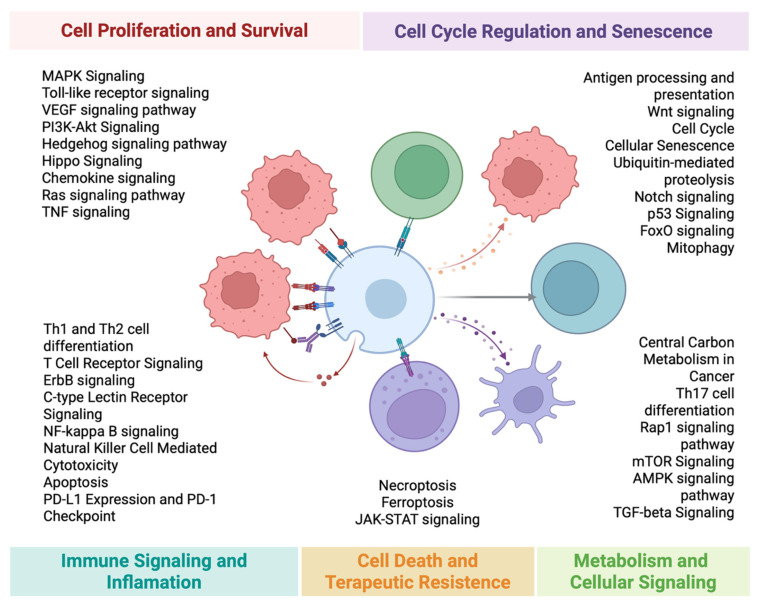
Overview of the key signaling pathways. Major biological processes are grouped into four functional domains: Cell Proliferation and Survival, Cell Cycle Regulation and Senescence, Immune Signaling and Inflammation, and Cell Death and Therapeutic Resistance. Figure 1 was created by the authors.

Somatic alterations (including pathogenic/likely pathogenic SNV/indel/CNV/fusion) were assigned to the predefined biological pathways (Figure 1). A pathway was considered “altered” if at least one gene in the pathway gene set harbored an eligible somatic alteration detected using the NGS platform.

### 2.5. Treatment Response Assessment

Response assessments were based on routine cross-sectional imaging performed in clinical practice, predominantly contrast-enhanced CT, with MRI used when clinically indicated. Imaging was performed per routine clinical practice, when available, every 8–12 weeks during ICI therapy; however, as a retrospective real-world cohort, the imaging schedules and assessment intervals were not uniform. Response was evaluated using RECIST v1.1 by institutional radiologists blinded to the molecular results.

### 2.6. Statistical Analysis

Continuous variables were assessed for distributional assumptions and summarized as mean (SD) when approximately normal or as median (IQR) when non-normal. Group comparisons used Welch’s *t*-test for normally distributed variables with unequal variances, Student’s *t*-test when variances were similar, and the Mann–Whitney U test for non-normally distributed variables. Categorical variables were compared using the χ^2^ or Fisher’s exact test, as appropriate. Exact two-sided *p*-values were reported.

Binary pathway alteration variables were compared between the TMB groups using two-sided Fisher’s exact tests because of sparse 2 × 2 tables. Odds ratios (ORs) with 95% confidence intervals and phi coefficients were reported as effect sizes for the analyses. Raw *p*-values are reported alongside the Benjamini–Hochberg false discovery rate (FDR)-adjusted *q*-values.

The primary endpoint was OS. Progression-free survival was not analyzed because of the limitations of retrospective capture, including heterogeneous imaging intervals and incomplete documentation of radiographic progression in routine practice. Analyses were restricted to ICI-treated patients with complete follow-up.

To mitigate the dilution of treatment-specific effects, OS was evaluated using two time origins: metastatic diagnosis and ICI initiation. Survival time was defined as the time from the relevant origin to death from any cause and was censored at the last follow-up. Kaplan–Meier curves were compared using the log-rank test. Cox proportional hazards models evaluated associations with prespecified covariates (age, clinical stage, MSI status, and TMB group); proportional hazards assumptions were assessed using Schoenfeld residuals. Results are reported as hazard ratios (HRs) with 95% confidence intervals.

As this was a retrospective cohort study with a fixed sample size, no a priori power calculations were performed. Analyses were hypothesis-generating and may have been underpowered for subgroup and pathway-level comparisons. Statistical analyses were performed using R 4.3.2 (R Foundation for Statistical Computing, Vienna, Austria). Statistical significance was defined as a two-sided *p* < 0.05. For pathway comparisons, FDR-adjusted *q*-values were used.

## 3. Results

From 1609 screened patients, 133 patients with advanced or metastatic tumors and TMB ≥ 10 mut/Mb underwent tumor genomic profiling using an extended DNA/RNA NGS platform. Patients were stratified into two prespecified categories based on TMB: 10–20 mut/Mb (TMB-H; *n* = 91) and >20 mut/Mb (TMB-VH; *n* = 42) (Table 1). The mean age at testing was 67 years, and 72/133 (54%) were male; age did not differ between the groups (Welch’s *t*-test *p* = 0.647). TMB values were right-skewed and are therefore presented descriptively; no hypothesis test was performed for TMB because it defined the comparison groups.

MSI was detected in 18/133 (14%) patients overall and was more prevalent in the TMB-VH group (38.1% vs. 2.2%; Fisher’s exact *p* = 8.9 × 10^−8^). The clinical stage at diagnosis, race, and diagnosis distributions were comparable between groups (Table 1). A detailed breakdown of the diagnoses is presented in Supplementary Table S1. The ICI regimens and response variables are listed in Supplementary Table S2.

**Figure 2:**
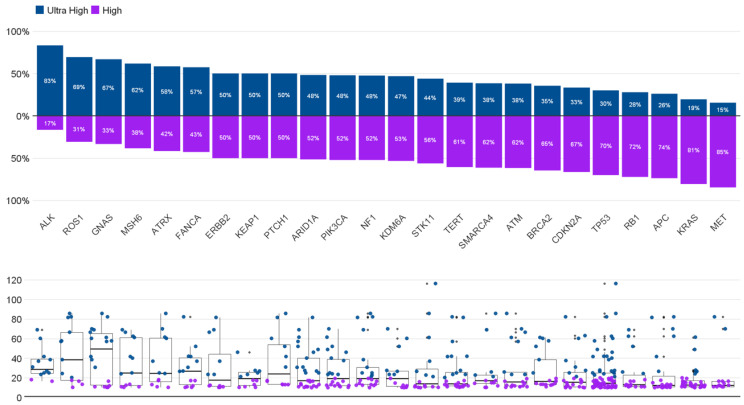
TMB distribution by recurrent gene alterations in the TMB ≥ 10 mut/Mb cohort. The upper panel shows the proportion of tumors classified as TMB-VH (>20 mut/Mb; blue) versus TMB-H (10–20 mut/Mb; purple) for each gene among cases harboring this alteration. The lower panel displays continuous TMB values by gene using box plots with individual data points.

To contextualize the extreme TMB at the gene level, we evaluated TMB distributions across recurrently altered genes (Figure 2). Several genes (e.g., GNAS, ROS1, and ALK) had a higher proportion of cases in the TMB-VH category, whereas others (e.g., KRAS and MET) were more frequently observed in the TMB-H category. Tumor suppressor alterations (e.g., TP53, RB1, and ATM) were observed in both strata. Figure 2 summarizes the dispersion of TMB values by gene context, including outliers in the hypermutated range.

**Figure 3:**
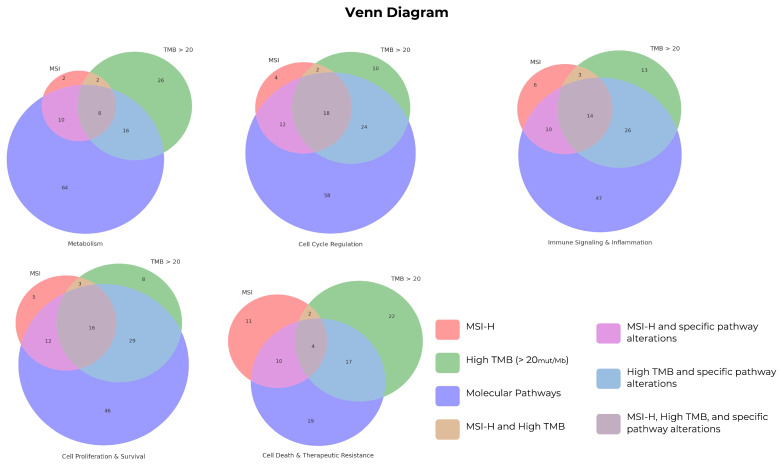
Overlap of pathway alteration categories by MSI and TMB statuses. Venn diagrams show the number of tumors with alterations in each pathway category (metabolism, cell cycle regulation, immune signaling and inflammation, cell proliferation and survival, and cell death/therapeutic resistance) among MSI-positive tumors, tumors with TMB > 20 mut/Mb, and their overlap. Counts represent the number of tumors that met each criterion and are presented as descriptive data.

Pathway-level comparisons did not identify statistically significant differences in alteration frequencies between the TMB groups after multiple testing corrections (all *q* > 0.05; Table 2). Several metabolic and signaling pathways showed numerically lower alteration frequencies in the TMB-VH group (including central carbon metabolism in cancer, C-type lectin receptor signaling, and JAK–STAT signaling) with small effect sizes (phi ≈ 0.06–0.18). Given the lack of significance after FDR correction, pathway-level results were reported descriptively (Table 2). Sensitivity analyses excluding MSI, POLE, and POLD1 somatic alterations yielded similar results. Additional pathway-level visualizations are shown in Figure 3 and Supplementary Figures S1–S4.

Among the 133 patients, 66 received at least one dose of ICI in the metastatic setting (Supplementary Table S2). Pembrolizumab was the most frequently used agent, followed by atezolizumab and ipilimumab/nivolumab. The objective response rate (complete or partial response) did not differ significantly between the TMB groups (Supplementary Table S2).

**Figure 4:**
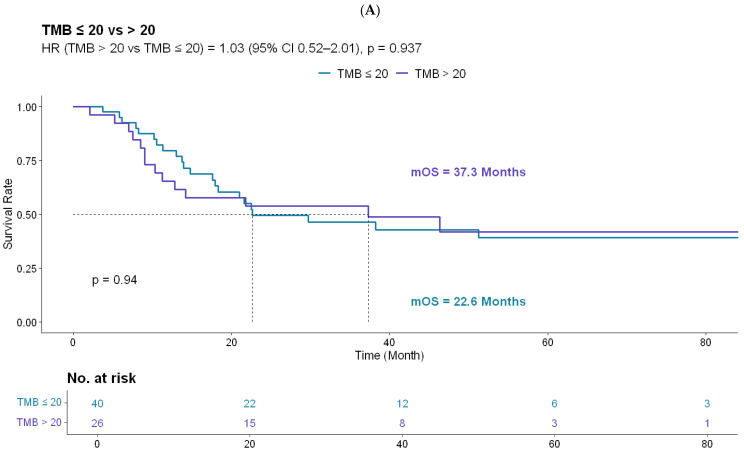

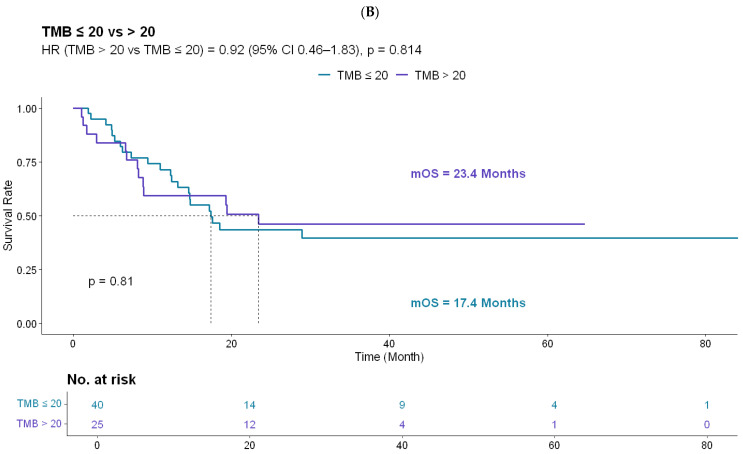
Overall survival (OS) by TMB status in the ICI-treated subset. Kaplan–Meier curves comparing OS between patients with TMB > 20 mut/Mb and those with TMB 10–20 mut/Mb using two time origins: (**A**) OS defined from the date of metastatic diagnosis (log-rank *p* = 0.937) and (**B**) OS defined from the date of ICI initiation (log-rank *p* = 0.814). The numbers at risk are shown below each panel.

To address potential lead-time bias related to the pre-ICI disease course, OS was evaluated in the ICI-treated subset using two clinically relevant time points. When defined from the metastatic diagnosis, OS did not differ significantly between the TMB groups (log-rank *p* = 0.937; HR 1.03, 95% CI 0.52–2.01) (Figure 4A). The median OS was 37.3 months for TMB-VH and 22.6 months for TMB-H (Figure 4A). When OS was defined from ICI initiation, the results were consistent, with no significant difference observed between the groups (log-rank *p* = 0.814; HR 0.92, 95% CI 0.46–1.83); the median OS was 23.4 months for TMB-VH and 17.4 months for TMB-H (Figure 4B).

In the multivariable Cox regression, clinical stage was independently associated with worse OS, whereas age, MSI status, and TMB group were not independently associated with OS. TMB-VH showed a non-significant trend toward improved survival (HR < 1) (Supplementary Figure S4).

## 4. Discussion

In this study, we examined the clinical outcomes and biological correlations of TMB in patients with advanced solid tumors by comparing TMB 10–20 mut/Mb with TMB > 20 mut/Mb. Two findings were most consistent in our dataset: MSI was substantially enriched among tumors with TMB > 20, and ICI-treated patients did not demonstrate a statistically significant OS advantage in the very high TMB group. Although Kaplan–Meier curves suggested numerically longer OS for TMB > 20 across both time origins (from metastatic diagnosis and ICI initiation), these differences were not statistically significant, and multivariable modeling did not identify the TMB category as an independent predictor of survival after adjustment for age, MSI status, and clinical stage.

These findings support a cautious and context-aware interpretation of TMB when applied as a stand-alone biomarker in heterogeneous real-world populations. Although TMB has been associated with ICI benefits at the population level in selected settings, its performance remains inconsistent across histologies, treatment lines, and sequencing platforms [12]. Tumor-agnostic application of fixed TMB thresholds risks oversimplifying the biological heterogeneity that affects immunogenicity and therapeutic sensitivity, including differences in tumor microenvironment composition, mutational processes, and tumor-intrinsic programs that shape antigen presentation and immune evasion [13,14]. In practical terms, a numeric TMB threshold captures only one dimension of a coupled immunobiological system in which the neoantigen burden, immune contexture, and tumor-intrinsic states collectively determine whether antigenicity translates into effective antitumor immunity.

The principal novelty of this study is the integration of TMB stratification with a pathway-level functional lens, emphasizing metabolic and signaling programs rather than treating TMB as a quantitative surrogate for the neoantigen load. No pathway-level differences remained statistically significant after multiple testing corrections, underscoring that these analyses are hypothesis-generating rather than confirmatory. This is particularly relevant because pathways are not independent units; they overlap, share genes, and converge on common nodes. True effects may be distributed across interconnected networks and diluted when each pathway is tested separately.

Despite the absence of FDR-significant pathway differences, we observed descriptive patterns in which several metabolic and signaling pathways appeared to be altered less frequently among tumors with TMB > 20. One biologically plausible (but unproven) interpretation is that extreme hypermutation may arise within distinct tumor-intrinsic contexts and under specific evolutionary constraints. Hypermutation driven by mismatch repair deficiency, polymerase proofreading defects, or exogenous mutagens may increase variant counts while simultaneously reshaping the selective pressures on growth, stress adaptation, and metabolism. In this framework, a lower alteration frequency in specific pathways could reflect alternative routes to tumor fitness, negative selection against additional disruption in highly unstable genomes, or residual histology-associated differences within TMB-defined strata. These signals require validation in cohorts designed to explicitly model the pathway interactions.

We also observed histology-associated variations in TMB-VH prevalence, consistent with prior reports that cancers linked to mutagenic exposures (e.g., melanoma and NSCLC) more frequently harbor extreme hypermutation [15]. Gastrointestinal malignancies contributed fewer TMB-VH cases in our cohort; however, this study was not designed to estimate population incidence and reflects real-world testing patterns and referral practices at a tertiary center. Nevertheless, these patterns underscore why a single TMB cutoff may perform differently across tumor types and why tumor-aware calibration and context-specific interpretation are likely necessary for clinical use.

Gene-level visualization further supports the biological heterogeneity within TMB-H. Some recurrent alterations were associated with broader TMB ranges and occasional hypermutated outliers, whereas others clustered within narrower distributions. Although our analysis was descriptive and not powered for gene-by-gene inference, these patterns support the broader concept that “TMB-H” encompasses biologically heterogeneous states and that the processes driving hypermutation differ across tumors. Future work should test whether combining TMB magnitude with genomic context (e.g., DNA repair alterations, polymerase proofreading defects, and MSI signatures) improves prediction of ICI benefit beyond a single numeric threshold [7,16].

A key implication is that improved prediction will likely require integrative models that combine correlated dimensions (TMB magnitude, MSI status, gene-level context, and pathway/network states) while accommodating nonlinear interactions and histology-specific priors. Artificial intelligence (AI) approaches may be valuable in this regard as well. Machine learning methods can integrate multi-omics and pathway-level features, identify non-obvious associations, and model interactions that are difficult to pre-specify using conventional stratified analyses. In larger multicenter datasets, AI-enabled models could incorporate mutational signatures and DNA repair states, pathway/network embeddings, and clinical features to generate more individualized predictions of ICI benefit and clarify whether and when TMB-VH adds predictive value beyond MSI and other covariates.

This study has limitations. First, the retrospective single-institution design introduces selection bias and limits generalizability. Second, given the size of the ICI-treated subset (*n* = 66; TMB-VH, *n* = 26), the study was likely underpowered to detect modest hazard ratios, increasing uncertainty around null findings, particularly for pathway-level comparisons. Third, our pathway framework simplifies interdependent biological networks and may miss context-dependent interactions. Fourth, germline testing was not uniformly available, limiting the assessment of hereditary predisposition and its contribution to the hypermutation. Fifth, OS follow-up was relatively short for a subset of patients; accrual was extended through August 2023, and follow-up was censored in January 2025, leaving several patients with approximately 12–18 months of survival observation, limiting the precision of long-term estimates. Finally, treatment exposure and line of therapy were heterogeneous, and residual confounding was likely, despite multivariable adjustment. Progression-free survival was not analyzed because the imaging intervals and progression ascertainment were heterogeneous in this retrospective cohort; therefore, OS was prioritized as the most consistently captured time-to-event endpoint.

Despite these constraints, the contribution of this study is not to establish a definitive predictive utility for extreme TMB thresholds but to provide a real-world, pathway-oriented framework that generates testable hypotheses regarding the biological context of hypermutation. The inclusion of a histologically diverse cohort spanning more than 20 tumor types and multiple ICI regimens enhances the relevance of this study to contemporary precision oncology. Larger multicenter cohorts with standardized sequencing, harmonized TMB definitions, and more uniform treatment annotations are required to validate these descriptive patterns and clarify their clinical utility. Future studies should evaluate integrative biomarker models incorporating TMB alongside MSI status, gene-level context, and pathway-level features to refine ICI selection [11] and test whether metabolic and signaling programs linked to hypermutation can inform rational combination strategies in appropriately designed prospective studies.

## 5. Conclusions

In this single-center, hypothesis-generating, real-world cohort study, TMB-VH captured biologically heterogeneous states and was strongly enriched for MSI. Although no pathway-level comparison remained significant after FDR correction, the descriptive patterns in metabolic and signaling pathways motivate further investigation of the functional context alongside TMB. Future multicenter studies should validate these findings and test integrative biomarker frameworks to better predict the benefits of immune checkpoint inhibitors.

## Figures and Tables

**Table 1:** Baseline clinical and demographic characteristics of the patients stratified by TMB status. Values are presented as mean (SD) for continuous variables and *n* (%) for categorical variables (percentages calculated within each TMB group). Age was compared using Welch’s *t*-test. MSI status was compared using Fisher’s exact test. Sex, race, and clinical stage were compared using χ^2^ tests with simulated *p*-values (5000 replicates) when indicated. The diagnosis *p*-value refers to the full multi-category diagnosis distribution (Supplementary Table S1). No hypothesis test was performed for TMB because it was used to define the comparison groups. * The complete diagnostic breakdown is provided in Supplementary Table S1. The ICI regimens and response variables are listed in Supplementary Table S2.

Variable	TMB-H (10–20, N = 91)	TMB-VH (>20, N = 42)	*p* -Value
**TMB (MUT/MB)**			-
Median (IQR)	12 (11–14)	38 (25–60)	
Range	10–20	20–116	
**Age**			0.647
Mean ± SD	67.8 ± 11.0	66.6 ± 15.0	
Range	43–97	27–94	
**Sex**			0.075
Female	47 (51.6%)	14 (33.3%)	
Male	44 (48.4%)	28 (66.7%)	
**MSI**			8.9 × 10^−8^
Positive	2 (2.2%)	16 (38.1%)	
Negative	88 (96.7%)	26 (61.9%)	
Indeterminate	1 (1.1%)	0 (0%)	
**Clinical stage at diagnosis**			0.735
I	1 (1.1%)	2 (4.8%)	
II	18 (19.8%)	9 (21.4%)	
III	13 (14.3%)	5 (11.9%)	
IV	35 (38.5%)	17 (40.5%)	
Unknown	24 (26.4%)	9 (21.4%)	
**Race**			0.545
White	33 (36.3%)	21 (50.0%)	
Asian	2 (2.2%)	1 (2.4%)	
Mixed	2 (2.2%)	1 (2.4%)	
Not declared	54 (59.3%)	19 (45.2%)	
**Diagnosis** *			0.515
Colorectal adenocarcinoma	8 (8.8%)	4 (9.5%)	
Urothelial carcinoma	9 (9.9%)	4 (9.5%)	
Melanoma	3 (3.3%)	4 (9.5%)	
Non-small cell lung cancer	28 (30.8%)	8 (19.0%)	
Other	43 (47.3%)	22 (52.4%)	

**Table 2:** Frequency of somatic alterations in selected biological pathways among tumors with TMB 10–20 vs. >20 mut/Mb. The pathway alteration variables were binary (present or absent). Comparisons between the TMB groups (10–20 vs. >20 mut/Mb) were performed using two-sided Fisher’s exact tests; odds ratios (OR) with 95% confidence intervals are shown. Phi denotes the effect size (ES). *p*-values are exploratory; q-values are Benjamini–Hochberg false discovery rates. All pathways had FDR-adjusted *q*-values > 0.05 (many rounding to 1.00), consistent with limited power for multiple comparisons.

Pathway	TMB 10–20 (N = 91)	TMB > 20 (N = 42)	OR (95% CI)	PHI	*p*	Q (FDR)
**Metabolism and general cell signaling**						
Central carbon metabolism in cancer	46/91 (50.5%)	13/42 (31.0%)	0.44 (0.20–0.95)	0.18	0.040	1.00
TH17 cell differentiation	24/91 (26.4%)	11/42 (26.2%)	0.99 (0.43–2.27)	0.00	1.000	1.00
RAP1 signaling pathway	14/91 (15.4%)	8/42 (19.0%)	1.29 (0.50–3.37)	0.05	0.621	1.00
MTOR signaling	41/91 (45.1%)	17/42 (40.5%)	0.83 (0.39–1.74)	0.04	0.708	1.00
AMPK signaling pathway	9/91 (9.9%)	1/42 (2.4%)	0.22 (0.03–1.81)	0.13	0.169	1.00
TGF-beta signaling	29/91 (31.9%)	12/42 (28.6%)	0.86 (0.38–1.91)	0.03	0.840	1.00
**Cell cycle regulation and senescence**						
WNT signaling	18/91 (19.8%)	6/42 (14.3%)	0.68 (0.25–1.85)	0.07	0.628	1.00
Cell cycle	47/91 (51.6%)	19/42 (45.2%)	0.77 (0.37–1.61)	0.06	0.577	1.00
Cellular senescence	49/91 (53.8%)	20/42 (47.6%)	0.78 (0.37–1.62)	0.06	0.577	1.00
Ubiquitin mediated proteolysis	11/91 (12.1%)	6/42 (14.3%)	1.21 (0.42–3.53)	0.03	0.782	1.00
NOTCH signaling	20/91 (22.0%)	10/42 (23.8%)	1.11 (0.47–2.64)	0.02	0.826	1.00
P53 signaling	41/91 (45.1%)	15/42 (35.7%)	0.68 (0.32–1.44)	0.09	0.349	1.00
FOXO signaling	49/91 (53.8%)	19/42 (45.2%)	0.71 (0.34–1.48)	0.08	0.456	1.00
Mitophagy	20/91 (22.0%)	10/42 (23.8%)	1.11 (0.47–2.64)	0.02	0.826	1.00
**Immune signaling and inflammation**						
Antigen processing and presentation	20/91 (22.0%)	11/42 (26.2%)	1.26 (0.54–2.94)	0.05	0.661	1.00
PD-L1 expression and PD-1 checkpoint	45/91 (49.5%)	19/42 (45.2%)	0.84 (0.41–1.76)	0.04	0.711	1.00
TH1 and TH2 cell differentiation	21/91 (23.1%)	10/42 (23.8%)	1.04 (0.44–2.47)	0.01	1.000	1.00
T cell receptor signaling	42/91 (46.2%)	16/42 (38.1%)	0.72 (0.34–1.52)	0.08	0.453	1.00
ERBB signaling	21/91 (23.1%)	7/42 (16.7%)	0.67 (0.26–1.72)	0.07	0.496	1.00
C-type lectin receptor signaling	37/91 (40.7%)	11/42 (26.2%)	0.52 (0.23–1.16)	0.14	0.123	1.00
NF-kappa B signaling	14/91 (15.4%)	7/42 (16.7%)	1.10 (0.41–2.96)	0.02	1.000	1.00
Natural killer cell mediated cytotoxicity	43/91 (47.3%)	17/42 (40.5%)	0.76 (0.36–1.59)	0.06	0.574	1.00
Chemokine signaling	34/91 (37.4%)	15/42 (35.7%)	0.93 (0.44–1.99)	0.02	1.000	1.00
**Cell proliferation and survival**						
MAPK signaling	45/91 (49.5%)	18/42 (42.9%)	0.77 (0.37–1.60)	0.06	0.576	1.00
Toll-like receptor signaling	13/91 (14.3%)	3/42 (7.1%)	0.46 (0.12–1.72)	0.10	0.390	1.00
VEGF signaling pathway	20/91 (22.0%)	10/42 (23.8%)	1.11 (0.47–2.64)	0.02	0.826	1.00
PI3K-AKT signaling	45/91 (49.5%)	17/42 (40.5%)	0.70 (0.33–1.46)	0.08	0.356	1.00
Hedgehog signaling pathway	5/91 (5.5%)	2/42 (4.8%)	0.86 (0.16–4.62)	0.02	1.000	1.00
HIPPO signaling	13/91 (14.3%)	6/42 (14.3%)	1.00 (0.35–2.84)	0.00	1.000	1.00
RAS signaling pathway	20/91 (22.0%)	10/42 (23.8%)	1.11 (0.47–2.64)	0.02	0.826	1.00
TNF signaling	13/91 (14.3%)	3/42 (7.1%)	0.46 (0.12–1.72)	0.10	0.390	1.00
**Cell death and therapeutic resistance**						
Apoptosis	28/91 (30.8%)	12/42 (28.6%)	0.90 (0.40–2.01)	0.02	0.842	1.00
Necroptosis	20/91 (22.0%)	11/42 (26.2%)	1.26 (0.54–2.94)	0.05	0.661	1.00
Ferroptosis	20/91 (22.0%)	10/42 (23.8%)	1.11 (0.47–2.64)	0.02	0.826	1.00
JAK-stat signaling	21/91 (23.1%)	5/42 (11.9%)	0.45 (0.16–1.29)	0.13	0.162	1.00

## Data Availability

Data supporting the findings of this study are available from the corresponding author upon reasonable request. Owing to privacy and ethical restrictions, some clinical data may not be shared publicly.

## Supplementary Materials

The following supporting information can be downloaded at: https://www.mdpi.com/article/10.3390/biomedicines14030593/s1, Figure S1. Kaplan–Meier OS by somatic alteration status for selected genes. Each panel compares OS (de-fined from metastatic diagnosis) between patients with and without the indicated somatic alterations (mu-tant vs. wild type) within the ICI-treated subset with complete follow-up. *p*-values (log-rank) and sample sizes are shown in each panel. These analyses were exploratory and are presented in a descriptive manner. Figure S2 shows a heatmap of the gene–diagnosis associations for the selected cancer types. Color in-tensity represents the unadjusted p-values for each gene–diagnosis pair (darker shading indicates smaller *p* values). The tumor types shown include colorectal adenocarcinoma (ADCOL), lung adenocarcinoma (ADPUL), urothe-lial carcinoma (CAURO), and melanoma (MELAN). The results are exploratory and intended to visualize the pat-terns of association. Figure S3. TMB distributions by somatic alteration status within tumor types (selected pairs). Box-plots compare continuous TMB values between tumors with and without specific somatic alterations for each diag-nosis. Only gene–diagnosis pairs with unadjusted *p* < 0.05 are displayed. These comparisons are exploratory and illustrate the context-specific variations in TMB dispersion. Figure S4 shows the relationship between multivariate Cox regression and OS. The forest plot shows the hazard ratios (log scale) and 95% confidence intervals for TMB > 20 mut/Mb, MSI status, age, and clinical stage (cStage) in the ICI-treated subset with complete follow-up. The dashed line indicates HR = 1. Table S1. Full diagnosis distribution by TMB group (10–20 vs. >20 mut/Mb). Values are expressed as *n* (%) for each TMB group. The *p*-value corresponds to the overall comparison across all diagnostic categories (χ^2^ with simulated *p*-values, 5000 replicates). Table S2. Immunotherapy exposure, regimen, and best response in ICI-treated patients stratified by TMB group. Values are expressed as *n* (%) unless otherwise specified. For the regimen and best response categories, the per-centages were calculated among ICI-treated patients within each TMB group. “Not evaluable/missing” indicates patients who received ICI therapy but lacked a documented response assessment. CR, complete response; nCR, near-complete response; PR, partial response; SD, stable disease; PD, progressive disease.
