# Supplementary material for: Very High vs. High Tumor Mutational Burden Across Tumors: Real-World Associations with MSI, Pathway Features, and Immunotherapy Outcomes
Source: Biomedicines. 2026 Mar 6;14(3):593. doi: 10.3390/biomedicines14030593 (PMC13024517; doi:10.3390/biomedicines14030593)
Supplement: Supplementary file 1 [file biomedicines-14-00593-s001.zip › biomedicines-4147109-supplementary.pdf]

Supplementary materials

Unless otherwise specified, exploratory analyses in the Supplementary Figures report unadjusted p-values.

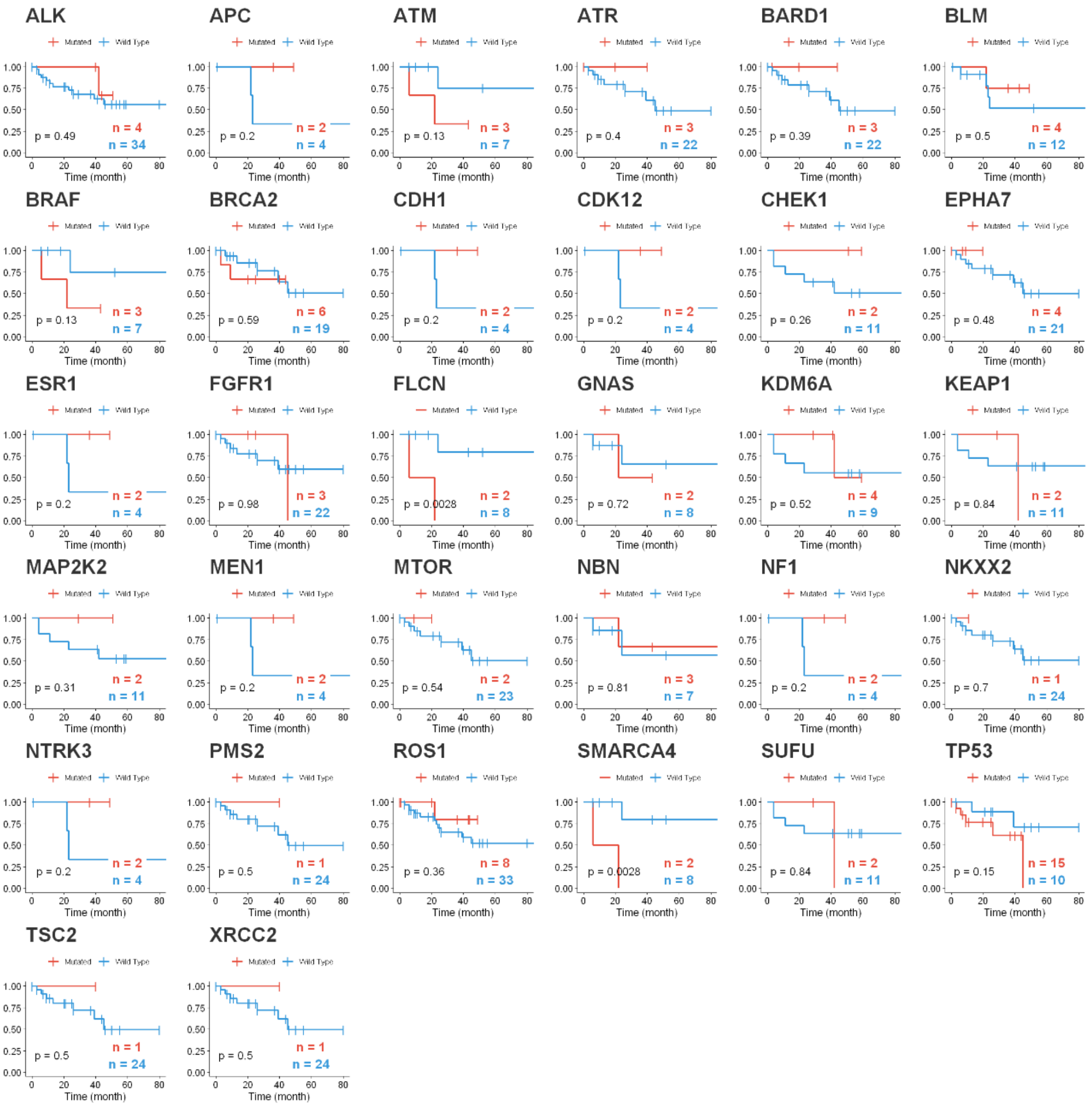

Supplementary Figure S1. Kaplan–Meier OS by somatic alteration status for selected genes. Each panel compares OS (defined from metastatic diagnosis) between patients with and without the indicated somatic alterations (mutant vs. wild type) within the ICI-treated subset with complete follow-up. P-values (log-rank) and sample sizes are shown in each panel. These analyses were exploratory and are presented in a descriptive manner.

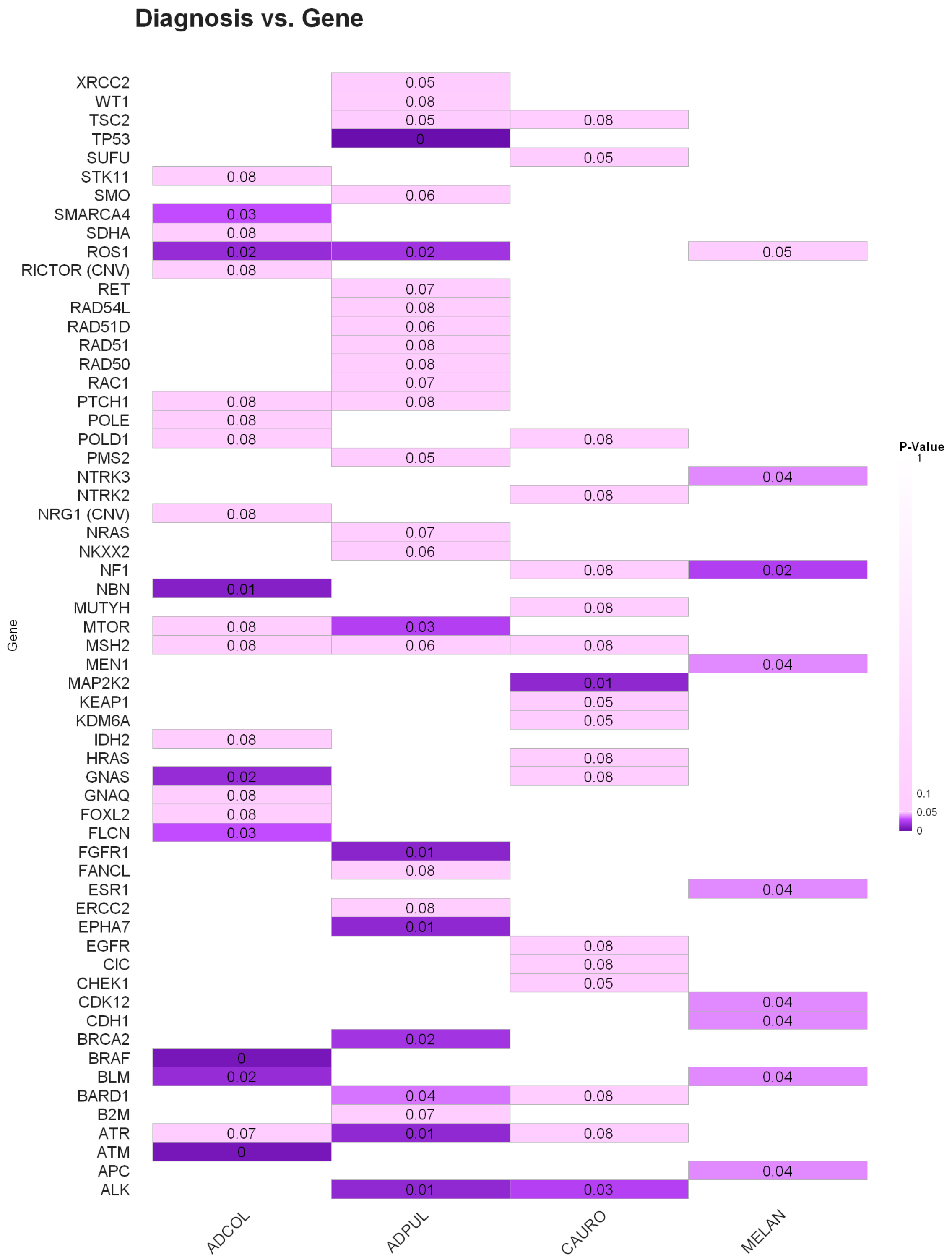

Supplementary Figure S2 shows a heatmap of the gene–diagnosis associations for the selected cancer types. Color intensity represents the unadjusted p-values for each gene–diagnosis pair (darker shading indicates smaller p values). The tumor types shown include colorectal adenocarcinoma (ADCOL), lung adenocarcinoma (ADPUL), urothelial carcinoma (CAURO), and melanoma (MELAN). The results are exploratory and intended to visualize the patterns of association.

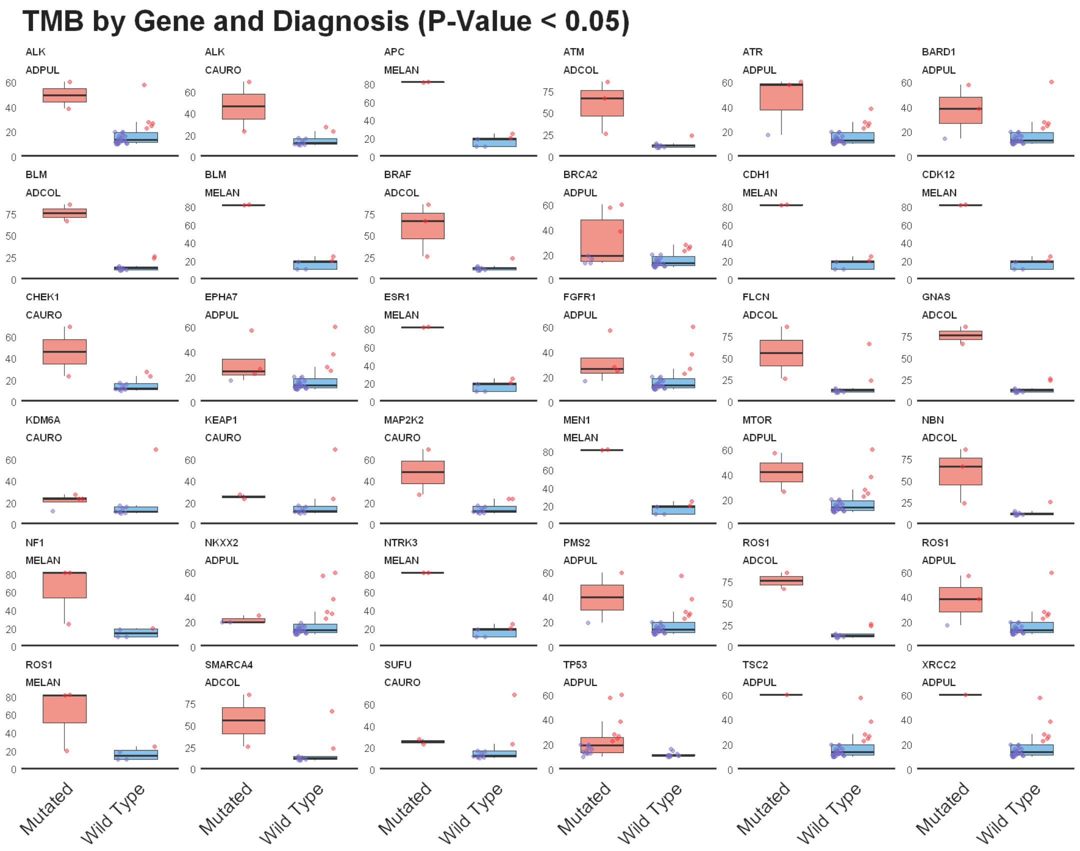

Supplementary Figure S3. TMB distributions by somatic alteration status within tumor types (selected pairs). Boxplots compare continuous TMB values between tumors with and without specific somatic alterations for each diagnosis. Only gene–diagnosis pairs with unadjusted p<0.05 are displayed. These comparisons are exploratory and illustrate the context-specific variations in TMB dispersion.

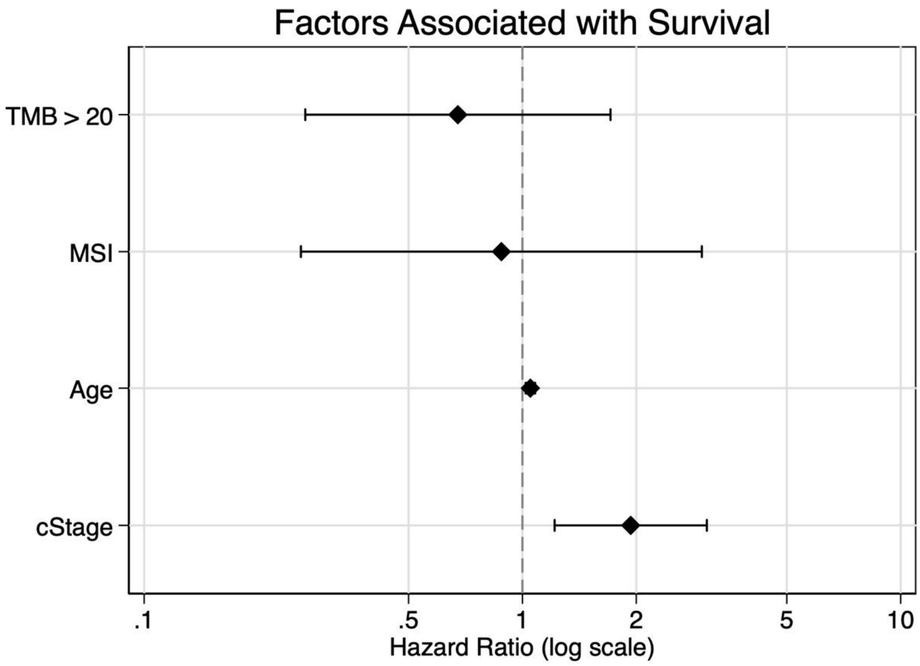

Supplementary Figure S4 shows the relationship between multivariate Cox regression and OS. The forest plot shows the hazard

ratios (log scale) and 95% confidence intervals for TMB >20 mut/Mb, MSI status, age, and clinical stage (cStage) in the ICI-treated subset with complete follow-up. The dashed line indicates HR=1.

Table S1. Full diagnosis distribution by TMB group (10–20 vs. >20 mut/Mb). Values are expressed as n (%) for each TMB group. The p-value corresponds to the overall comparison across all diagnostic categories (χ^2 with simulated p-values, 5,000 replicates).

HISTOLOGIC SUBTYPE	TMB-H (10-20, N=91)	TMB-VH (>20, N=42)
COLORECTAL ADENOCARCINOMA	8 (8.8%)	4 (9.5%)
ENDOMETRIAL ADENOCARCINOMA	2 (2.2%)	2 (4.8%)
PANCREATIC ADENOCARCINOMA	2 (2.2%)	1 (2.4%)
PROSTATE ADENOCARCINOMA	0 (0.0%)	2 (4.8%)
HEPATOCELLULAR CARCINOMA	1 (1.1%)	0 (0.0%)
NEUROENDOCRINE CARCINOMA	2 (2.2%)	1 (2.4%)
THYMIC CARCINOMA	1 (1.1%)	0 (0.0%)
BASAL CELL CARCINOMA	1 (1.1%)	2 (4.8%)
HEAD AND NECK CARCINOMA	2 (2.2%)	1 (2.4%)
BREAST CARCINOMA	6 (6.6%)	0 (0.0%)
OVARIAN CARCINOMA	3 (3.3%)	1 (2.4%)
SMALL CELL LUNG CANCER	3 (3.3%)	1 (2.4%)
THYROID CANCER	1 (1.1%)	0 (0.0%)
ESOPHAGOGASTRIC CARCINOMA	3 (3.3%)	2 (4.8%)
UNKNOWN PRIMARY SITE CARCINOMA	5 (5.5%)	1 (2.4%)
UROTHELIAL CARCINOMA	9 (9.9%)	4 (9.5%)
CHOLANGIOCARCINOMA	5 (5.5%)	1 (2.4%)
GLIOMA	1 (1.1%)	1 (2.4%)
LYMPHOMA	2 (2.2%)	2 (4.8%)
MELANOMA	3 (3.3%)	4 (9.5%)
**NON-SMALL CELL LUNG CANCER**	28 (30.8%)	8 (19.0%)
**UNKNOWN**	3 (3.3%)	4 (9.5%)

Table S2. Immunotherapy exposure, regimen, and best response in ICI-treated patients stratified by TMB group. Values are expressed as n (%) unless otherwise specified. For the regimen and best response categories, the percentages were calculated among ICI-treated patients within each TMB group. "Not evaluable/missing" indicates patients who received ICI therapy but lacked a documented response assessment. CR, complete response; nCR, near-complete response; PR, partial response; SD, stable disease; PD, progressive disease.

	TMB 10-20 (N=38)	TMB > 20 (N=26)
**IMMUNOTHERAPY TYPE**		
PEMBROLIZUMAB	26 (65.0%)	21 (80.8%)
ATEZOLIZUMAB	5 (12.5%)	2 (7.7%)
IPILIMUMAB + NIVOLUMAB	4 (10.0%)	2 (7.7%)

DURVALUMAB	2 (5.0%)	0 (0.0%)
NIVOLUMAB	2 (5.0%)	0 (0.0%)
AVELUMAB	1 (2.5%)	0 (0.0%)
CEMIPLIMAB	0 (0.0%)	1 (3.8%)
**BEST RESPONSE**		
CR/NCR	5 (13.1%)	6 (23.1%)
PR	17 (42.5%)	8 (30.8%)
SD	6 (15.0%)	5 (19.2%)
PD	10 (25.0%)	6 (23.1%)
**MISSING / NOT EVALUABLE**	2 (5.0%) *	1 (3.8%) *

***Patients who received ICI therapy but lacked documented radiologic response assessment.**
